# Towards an integrative structural model of the fishnet slit diaphragm: Understanding its physiology through proteomics and structural insight

**DOI:** 10.1007/s00424-026-03200-0

**Published:** 2026-07-22

**Authors:** Achilleas S. Frangakis, Alexandra N. Birtasu, Florian Grahammer

**Affiliations:** 1 Buchmann Institute for Molecular Life Sciences, Institute of Biophysics, Goethe University Frankfurt, 60438 Frankfurt, Germany; 2 III. Department of Medicine, University Medical Center Hamburg-Eppendorf, 20246 Hamburg, Germany; 3 Hamburg Center for Kidney Health (HCKH), University Medical Center Hamburg- Eppendorf, 20246 Hamburg, Germany

## Abstract

The kidney slit diaphragm (SD) connects the foot processes of glomerular podocytes, anatomically forming the outermost part of the three-layered glomerular filtration barrier. The SD is a large molecular polymer that, together with the foot processes, covers the glomerular capillaries and has diverse functions. It establishes a strong yet flexible cell-cell contact between foot processes from neighboring podocytes and additionally acts as a dynamic signaling platform linking structural integrity with cellular responses. Understanding the physiology of the SD has been hampered by limited structural insights and a lack of precise knowledge of protein composition of its building blocks.

The recent convergence of the proteomic analyses of the SD with atomically resolved structures and in situ cryo-electron tomography imaging have led to the fishnet SD. The fishnet SD is based on the high-resolution 3D cryo-electron tomographic images of native glomeruli that revealed an unexpected fishnet architecture, observed in mouse, fly and human. This breakthrough has provided a new and markedly different view of how we understand and analyze the SD. It provides important principles for understanding spatial self-organization, assembly, turnover, and force compensation and offers an important basis for functional investigations, including the elucidation of molecular mechanisms of clinically relevant diseases.

## Introduction to the glomerular filtration barrier

The kidney is responsible for filtering blood and maintaining fluid and electrolyte balance. Its functional units are the nephrons. At the tip of each nephron, the glomerulus - a tuft of a specialized capillary bed - is the site where filtration of blood takes place. These capillaries with their fenestrated endothelial cells form the innermost part of the three-layered glomerular filtration barrier (GFB). The second layer is the glomerular basement membrane (GBM) which provides a matrix ensuring a cell-free and size-selective filtration of plasma. The outermost layer consists of podocytes, large octopus-like cells which cover the largest part of the GBM. Their interdigitating foot processes form narrow gaps bridged by the slit diaphragm (SD), which anatomically represents the final filtration layer. Together, the three-layered GFB allows the passage of small molecular weight substances like water, electrolytes, glucose, and urea into Bowman’s space while retaining high molecular weight components and cells in the blood [20]. Damage to this barrier, as seen in conditions such as nephrotic or nephritic syndrome, disrupts this selectivity and can lead to the leakage of proteins or even blood cells into the urine [4].

Elucidating the molecular composition and structure of the GFB has been a long-sought goal addressing essential questions regarding: (i) the molecular mechanism underlying the filtration process, (ii) how the filtration barrier avoids clogging over an average human lifespan of eight decades, and (iii) how changes in filtration or primary filtrate composition are communicated to the cells. Furthermore, reduced kidney function is frequently associated with a breakdown of the GFB. Within the GFB, the SD plays a central role in maintaining filtration selectivity. Accordingly, SD dysfunction and pathological changes to the SD have a high pathomechanistic relevance in acquired and hereditary renal diseases that lead to proteinuria, such as minimal change disease, focal-segmental glomerulosclerosis, and diabetic nephropathy [1]. A deep and thorough understanding of SD composition and structure is essential to better understand disease processes on a molecular level. Finally, proteinuria has long been recognized as an independent risk factor for renal and cardiovascular disease [3], and novel targets to halt or even reverse proteinuria are highly sought after.

In this review, we discuss the latest developments that have led to the characterization of the fishnet SD. Cryo-electron tomography has provided three-dimensional images of the SD, demonstrating unequivocally its fishnet-like architecture. In parallel, proteomic analyses have determined the stoichiometries of the SD protein constituents, which, in combination with available X-ray crystallographic data, have enabled the fitting of the SD constituents into the densities of the cryo-electron tomograms. We also address the current limitations of the data, as the restricted resolution does not yet permit detailed analysis of the precise intra- and extracellular interaction interfaces and hence the proposed molecular fishnet models will be refined and improved in the future. Finally, we argue that further data acquisition will improve resolution and ultimately allow for the elucidation of the specific interactions that define the SD’s function and properties.

## Proteomics analysis provides the interaction network of the SD constituents

Modern proteomics technologies applied to affinity-isolated SDs from rat and mouse glomeruli allowed for the understanding of the molecular composition of SD building blocks. A network of approximately 30 proteins that interact with both Nephrin and Neph1 - the main constituents of the SD - was established [10]. A substantial fraction of these interacting proteins are type-1 transmembrane proteins, several of which contain extracellular Ig or Ig-like domains. Functionally, the interactome constituents cover different cellular functions including signaling at and across the plasma membrane, cell adhesion, and scaffolding. Interestingly, some of them are known for their involvement in synapse formation in the brain (PTPRO, MAGI1, MAGI2, Robo2, Dendrin, APP and A4-ITM2B) [9, 12–15, 22]. While the interactome provided a number of expected hits, the majority of the identified proteins have not previously been reported to interact with either Nephrin or Neph1. Functional studies support their relevance: Knock-down of these proteins in the *Drosophila melanogaster* model system profoundly reduced albumin uptake or atrial natriuretic peptide accumulation, demonstrating an impact on SD function. Another prominent protein at the SD that was thought to be closely associated with Nephrin and Neph1 is Podocin. However, the Podocin interactome differed substantially from that of Nephrin and Neph1, reflecting the dual localization of this protein serving as a cytoplasmic anchor for a series of SLC-type and other transporters in the plasma membrane [10].

An emerging body of research has shed light on changes in the structure, stability and dynamic remodeling of the SD networks that are induced following genetic deletion of its core components Nephrin, Neph1, and Podocin [10]. Three findings should be highlighted. First, structural and functional integrity of the SD networks particularly rely on Neph1. Reduced Neph1 levels correlated with a rise in albuminuria observed in knock-out (KO) models of all three core constituents, even in Nephrin KO mice, where overt albuminuria appears only after 12 weeks and is accompanied by a decline in Neph1 content. Second, the network constituents that directly assemble with the Nephrin–Neph1 core are Dendrin, MAGI2, PTPRO, and FYN [9, 12–15, 22]. Functionally, MAGI2 is a scaffolding protein, while PTPRO is a tyrosine phosphatase and FYN a tyrosine kinase, which underscores the relevance of tyrosine phosphorylation events occurring within slit proteins, especially Nephrin. The role of Dendrin within the SD remains unclear, despite its proposed involvement in apoptotic processes of podocytes and neurons. Third, the assembly of the SD network is regulated by protein phosphorylation. Accordingly, phosphorylation at an acidic cluster (simultaneous modification of three Serine/Threonine residues) immediately adjacent to the transmembrane domain stabilizes network-integration of Nephrin, whereas its dephosphorylated form is readily degraded after Nephrin-KO induction [10].

Altogether the SD proteomic analysis centered around the core constituents, Nephrin, Neph1 and Podocin shows a tight link to the cytoskeleton and the cytoplasmic signaling environment [10]. Stunningly, such a network is reminiscent of the proteinaceous bridges formed between the pre- and postsynaptic membranes of synapses in the central nervous system by the cleft-spanning single transmembrane-domain proteins Neurexins and Neuroligins and their associated downstream networks [21]. With the identification of the core SD protein components, new avenues for investigating their precise roles and functions have opened. In particular, protein interactions, complex assembly, signaling, and dynamic tyrosine phosphorylation position the SD as a ‘signaling hub’ in the filtration barrier need to be further explored. An emerging field of research will be the interplay between SD composition and its structural backbone formed by the Nephrin-Neph1 fishnet architecture, which will be further explored in the subsequent paragraph.

### Structural insights into native slit diaphragm organization

**Fig. 1:**
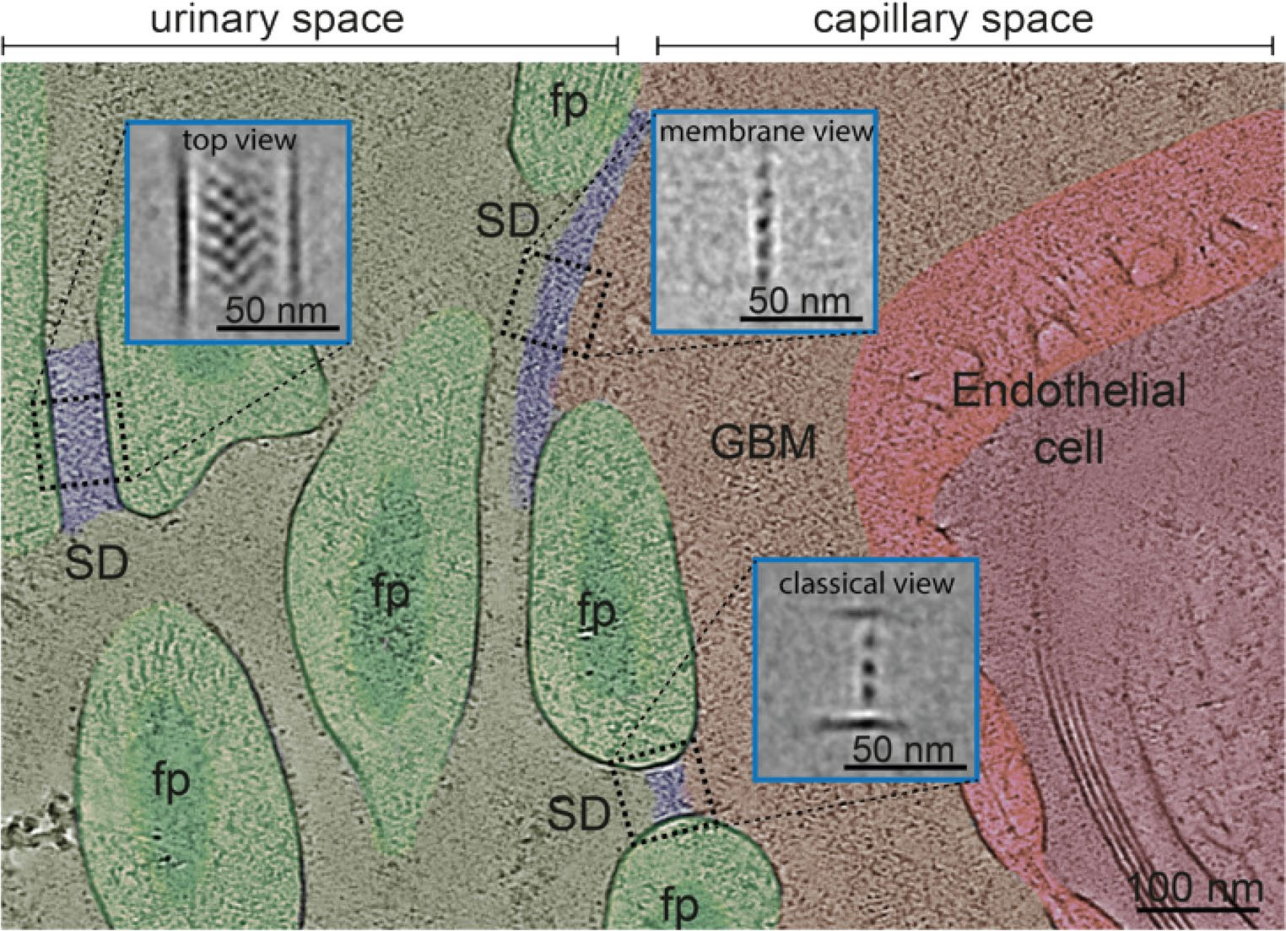
Structural organization of the glomerular filtration barrier. Slice through a cryo-electron tomogram of the tripartite organization of the glomerular filtration barrier. Pseudo-colors facilitate the interpretation of spatial organization, highlighting key components such as the podocyte foot processes (green) bridged by the slit diaphragm (blue) in the urinary space, the glomerular basement membrane (brown), and in the capillary space the fenestrated glomerular endothelial cells (magenta). This allows contextual information on the native ultrastructure within the glomerulus and provides orthogonal views of the slit diaphragm (view direction is indicated – insets adapted from [2]

Cryo-electron tomograms of mice, *Drosophila*, and human samples reveal an SD with an evolutionarily conserved fishnet-like architecture (Fig. 1) [2, 16, 17]. The spacing between podocyte foot processes ranges from 44 to 53 nm across species, and the strands of the fishnet SD spanning the adjacent podocytes (Fig. 1) are spaced approximately 9 nm apart in humans, 12 nm in mice, and 15 nm in *Drosophila*. Genetic differences are likely responsible for species-specific variations of strand crossings within the extracellular space. This experimentally validated arrangement of intercellular molecules represents a paradigm shift beyond earlier, largely hypothetical SD models [7].

**Fig. 2:**
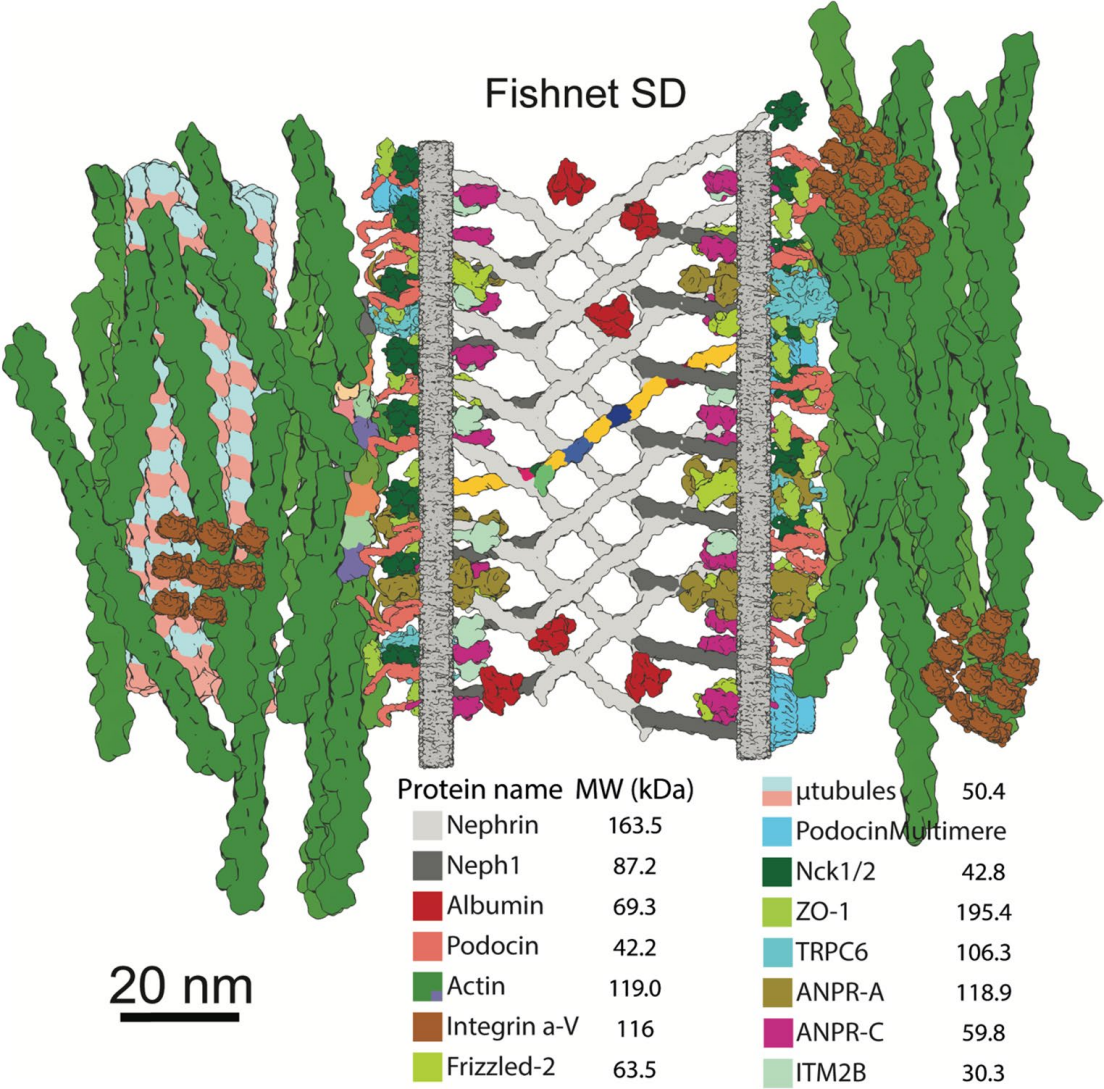
Integrative model of the fishnet slit diaphragm showing the molecular architecture based on structural data combined with proteins identified by proteomic analyses. All proteomics-based mapped components are positioned within the SD framework, illustrating how high-resolution structural information can be complemented by orthogonal datasets, such as proteomics, to build a comprehensive interaction hub and to better understand the functional organization of the filtration barrier. Membranes of adjacent foot processes are shown with several protein components of the SD (color coded). The predicted interaction surfaces between Nephrin-Neph1 molecules are highlighted by color. Proteins, respective genes, and molecular weight are indicated. ZO-1 (zonola occludens-1), TRPC6 (Transient receptor potential cation channel subfamily C member 6), ANPR-A /C (Atrial natriuretic peptide receptor A/C), ITM2B (Integral membrane protein 2B) [2, 10]

The combination of X-ray crystallographic structures, cryo-electron tomographic images and proteomics studies provide the direct interpretation that the strands of the fishnet SD are heterodimers of the cell-adhesion molecules Nephrin and Neph1. In this model, Nephrin and Neph1 criss-cross to generate a multitude of interactions. These form an extended micrometer-scale periodicity of the fishnet SD that is conserved across species, indicating the presence of (self-)organizing principles that facilitate polymer assembly. Importantly, the fishnet architecture is directly supported by imaging data, whereas the precise molecular interactions underlying this architecture have been modeled and remain subject to further structural analysis and physiological validation (Fig. 2).

## Towards an integrative structural model of the fishnet SD

The discovery of the fishnet SD relied on cryo-electron tomography, which - despite its similarity in name to conventional electron tomography and electron microscopy used in earlier studies - differs markedly in sample preparation. In cryo-electron tomography, samples are imaged under near-native conditions and averaged from hundreds to tens of thousands of individual pictures to increase the contrast and the signal-to-noise ratio [5]. In all previous SD studies, conventional electron microscopy was used, which typically involves harsh chemical fixation, embedding, and staining with heavy elements to enhance contrast. While this staining increases contrast, it comes at the cost of reduced resolution. Consequently, previous electron microscopy and tomography studies that led to the “zipper” [19], the “multilayered scaffold” [8] or the “porous slit diaphragm“ [6] models lacked the resolution required to resolve the fishnet architecture. Hence, the overall architecture of these models, which feature an array of linearly arranged adjacent Nephrin and Neph1 molecules, differs substantially from the fishnet SD and does not reflect the true architecture of the SD [7].

Despite significant progress, several questions remain unanswered to date. These include dynamic, time-resolved SD remodeling, molecular turnover, and mechanisms of disease progression. Typically, addressing those would require both high spatial and high temporal resolution, which cannot be addressed simultaneously and therefore require indirect approaches. Within the fishnet SD, one Nephrin–Neph1 heterodimer interacts with at least four neighboring heterodimers. Notably, up to three consecutive molecules could be removed without compromising adhesion or substantially altering pore spacing within the SD, overcoming a key limitation of the “zipper model”. However, it remains unclear how molecular replacement appears feasible within this framework. Specifically, how could a newly synthesized molecule, once trafficked to the surface of a podocyte foot process, readily integrate into the SD by binding to adjacent Nephrin–Neph1 heterodimers? The inverse process of a Nephrin molecule removal raises similar spatial-temporal questions. The overall fishnet SD is conserved among species despite genetic variations in the orthologues of Nephrin and Neph1. It is composed of one or more layers, each of them exhibiting the same basic fishnet architecture. How individual mutations affect the SD architecture is currently unclear. Interestingly, virtually all known mutations in Nphs1 to date prevent stable protein presentation on the plasma membrane, thereby completely preventing the formation of any fishnet architecture. Functional studies show that disruption of either core structural proteins or selected associated network components, including signaling-receptors, kinases/phosphatases, transporters and scaffolds, leads to specific defects in the filtration process.

For reaching an integrative structural model of the fishnet SD, further improvements in resolution are needed. At present (20–30 Å resolution), the exact molecular interfaces between criss-crossing strands are not yet fully defined. The molecular interactions can only be inferred indirectly, for example through molecular dynamics simulations, but ultimately require direct visualization for definitive validation. Currently, only one of up to four inferred interfaces has been experimentally verified. Ozkan et al. reported the crystallographic structure and heterophilic complex of the Nephrin and Neph1 homologs SYG-1 and SYG-2 in *C. elegans* and identified several amino acid interactions, including a key glutamine–glutamine interaction (Q53–Q54) and several other adjacent supporting interactions [18]. This SYG-1/SYG-2 interface occupies a central position in the integrative structural model of the Ig1-Ig1 interaction interface underlying the fishnet SD. Atomistic molecular dynamics simulations further support the stability of this interface across all three species examined, demonstrating its conservation despite species-specific genetic variation.

Verification of the remaining interfaces is considerably more challenging, as these interfaces are predicted to be less stable - consistent with molecular dynamics simulations - and may need to retain a degree of flexibility to allow for the dynamic properties and turnover behavior of the SD constituents. Thus, while the overall fishnet architecture constrains the possible locations of interaction sites, the precise molecular contacts, which likely differ across species because of the geometric variations, remain imprecisely defined. Mapping disease-associated mutations to these regions may help validate their functional relevance, but the extent to which individual variants perturb secondary structure or disrupt specific interaction interfaces requires further investigation. Precise identification of the interaction sites is therefore a prerequisite and will require high-resolution structural approaches. Only then can the effects of disease-associated mutations be rigorously assessed. Results from current state-of-the-art technologies indicate that achieving sub-10 Å resolution for the fishnet SD is within reach [11]. Reaching this level of detail will require additional cryo-electron tomograms of the fishnet SD to unambiguously resolve individual Ig domains and interaction sites.

## Data Availability

No datasets were generated or analysed during the current study.
